# Tropylium Ion, an Intriguing Moiety in Organic Chemistry

**DOI:** 10.3390/molecules28104095

**Published:** 2023-05-15

**Authors:** Fatima Tuz Zahra, Aamer Saeed, Khansa Mumtaz, Fernando Albericio

**Affiliations:** 1 Department of Chemistry, Quaid-i-Azam University, Islamabad 45320, Pakistan; fatimazahra@chem.qau.edu.pk (F.T.Z.); khansamumtaz1997@gmail.com (K.M.); 2 School of Chemistry and Physics, University of KwaZulu-Natal, Durban 4001, South Africa; 3 CIBER-BBN, Networking Centre on Bioengineering, Biomaterials and Nanomedicine, Department of Organic Chemistry, University of Barcelona, 08028 Barcelona, Spain

**Keywords:** tropylium cation, tropylium tetrafluoroborate, selectivity, electrophile, organo-catalyst, universal oxidant, Lewis acid catalyst, coupling reagent

## Abstract

The tropylium ion is a non-benzenoid aromatic species that works as a catalyst. This chemical entity brings about a large number of organic transformations, such as hydroboration reactions, ring contraction, the trapping of enolates, oxidative functionalization, metathesis, insertion, acetalization, and trans-acetalization reactions. The tropylium ion also functions as a coupling reagent in synthetic reactions. This cation’s versatility can be seen in its role in the synthesis of macrocyclic compounds and cage structures. Bearing a charge, the tropylium ion is more prone to nucleophilic/electrophilic reactions than neutral benzenoid equivalents. This ability enables it to assist in a variety of chemical reactions. The primary purpose of using tropylium ions in organic reactions is to replace transition metals in catalysis chemistry. It outperforms transition-metal catalysts in terms of its yield, moderate conditions, non-toxic byproducts, functional group tolerance, selectivity, and ease of handling. Furthermore, the tropylium ion is simple to synthesize in the laboratory. The current review incorporates the literature reported from 1950 to 2021; however, the last two decades have witnessed a phenomenal upsurge in the utilization of the tropylium ion in the facilitation of organic conversions. The importance of the tropylium ion as an environmentally safe catalyst in synthesis and a comprehensive summary of some important reactions catalyzed via tropylium cations are described.

## 1. Introduction

The tropylium ion is a cyclic, planar organic cation with the chemical formula C_7_H_7_^+^. It is formed by the protonation of tropine, which is a bicyclic organic compound found in plants such as *Atropa belladonna* and *Hyoscyamus niger*.

The tropylium ion has six π-electrons in its conjugated ring system, which gives it aromatic properties. The structure of the ion is reminiscent of the cycloheptatrienyl anion (tropylium’s isoelectronic counterpart) and is stabilized by the delocalization of the positive charge over the seven carbon atoms of the ring.

The tropylium ion is an important intermediate in the synthesis of many alkaloids, such as atropine and cocaine. It is also used as a spectroscopic probe in organic chemistry research.

The generation of stable and neutral aromatic systems [1] such as benzenoid frameworks [2] has been the driving force [3] for innumerable organic reactions [4] in synthetic chemistry [5]. Their lesser-known analogs, the non-benzenoid [6] carbocyclic aromatic structures [7], used to be considered far less synthetically valuable. These fundamental chemical species [8] usually possess a net charge [9] and are likely to be less stable than benzenoid systems [10].

Merling’s discovery of the bromide salt of the cycloheptatrienyl cation [11], also known as the tropylium ion (**1b**), in 1891 led to the creation of non-benzenoid carbocyclic aromatic ions [12]. The tropylium ion (**1b**) is a seven-membered aromatic ring [13] (C_7_H_7_^+^) utilized in chemical synthesis [14] and as a metal complex ligand [15]. The non-benzenoid aromatic tropylium ion [16] (**1b**) has a 6π-electron system and possesses a positive charge entirely delocalized on a conjugated planar seven-carbon system [17]. As a result, it satisfies Hückel’s aromaticity [18] rule and has a unique combination of stability and reactivity [19].

**Scheme 1:**
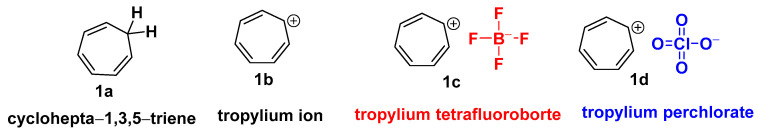
Structural representation of tropylium ion and its salts (**1c**) and (**1b**).

Nguyen’s research group has made significant contributions to tropylium ion chemistry [20]. He reasoned that the carbocation with one positive charge delocalized [21] over the conjugated seven-membered ring potentially serves as a “soft Lewis catalyst” [22]. Salts of this ion are known to be relatively stable in the solid state or in solutions [23]. It is used in reactions in the form of various salts, such as tropylium tetrafluoroborate (**1c**) and tropylium perchlorate (**1d**) (Scheme 1).

This aromatic ion can be reversibly converted to its non-aromatic neutral structure form by associating with the counterion. As the generation of stable aromatic systems can provide the driving force for many types of chemical transformations, it was believed that the equilibrium between the tropylium ion (**1b**) and its neutral form could play a vital role in the promotion of organic reactions [24]. Recently, the idea of utilizing this system to facilitate chemical reactions has evolved into versatile methodologies. To date, there have been numerous reports on the applications of non-benzenoid aromatic tropylium ions to accelerate chemical reactions [25].

Due to the reactivity of charged systems, (**1b**) is capable of forming a covalent bond with other substrates to produce reactive intermediates for further chemical transformations [26]. The reactive intermediates tend to re-aromatize, hence providing the driving force for chemical reactions [27]. The unique combination of stability (due to aromaticity) and reactivity (due to the associated net charge) equips tropylium ions with the unique capability to promote chemical processes [28]. The mechanism of activation by the tropylium ion is potentially applicable to an extended range of substrates and reactions [29]. Furthermore, it can also be used as an organocatalyst, as its neutral de-aromatized counterparts can often be easily converted back to the original aromatic form in situ by the addition of activating reagents [30]. Recently, metal catalysts have been replaced by organocatalysts [31] owing to better tolerance to moisture and air, as well as excellent compatibility with a diverse variety of substrate functional groups [32] and the contribution to green chemistry. This review focuses on the recent roles of the tropylium (positively charged, 7C, 4n + 2 = 6π-electrons) ion in synthetic chemistry [33].

Tropylium ions (**1b**) and their salts can be synthesized by a variety of different methods.

Under optimal conditions, the reaction of 4-substituted-4-pentenal derivatives (**2a**) with (**2b**) synthesized from pyruvic ester produced 1,6-di- and 1,6,7-trisubstituted-1,3,5-cycloheptatrienes (**A**) in reasonable yields. The synthesis is attractive since it involves a broad substrate scope (**i**–**x**), commercially available chemicals, has milder reaction conditions than previously reported, and requires minimal manipulation [34]. Heptamethyltropylium perchlorate (**B**) has been synthesized via the reaction of 1,2,3,4,5,6,7-heptamethyltropilidene (**3a**) with phosphorus pentachloride and subsequent treatment with perchloric acid [35].

**Figure 1:**
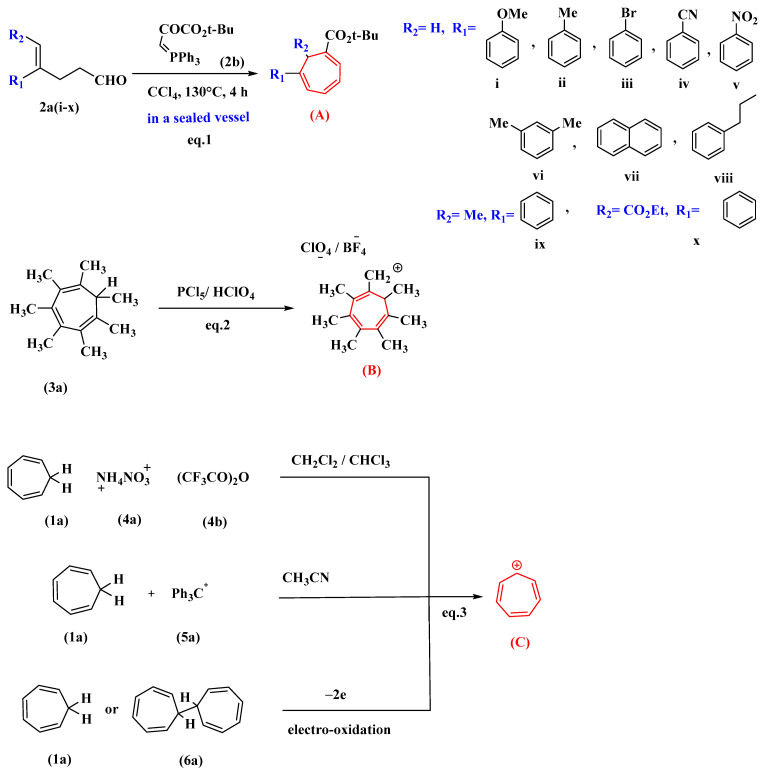
Various methods for synthesizing tropylium salt (**C**) and its derivatives (**A**,**B**). Di and trisubstituted cycloheptatrienes (**A**) synthesized from pyruvic ester (**2a**) is shown in **eq.1**. Heptasubstituted cycloheptatriene (**B**) synthesized from heptamethyltropilidene (**3a**) is shown in **eq.2**. Tropylium salts (**C**) synthesized from cycloheptatriene (**1a**) is shown in **eq.3**.

Tropylium ion salts **(C)** are prepared by the oxidation of cycloheptatriene (**1a**) with ammonium nitrate (**4a**) and trifluoroacetic anhydride (**4b**). This reaction proceeds at room temperature in chloroform or methylene chloride. The reaction completion is indicated by the red solution of tropylium trifluoroacetate salt, but this salt cannot be isolated, so it is converted to other stable salts by the addition of suitable acids in the reaction mixture in the presence of ethanol as a solvent [36]. In the presence of acetonitrile, a hydride exchange reaction between cycloheptatriene (**1a**) and tritylium salts (**5a**) produced tropylium salt (**1c** or **1d**) [37]. Tropylium ions can also be synthesized by the electro-oxidation of tropilidene, the “parent” hydrocarbon (**1a**). The electrolysis medium used was acetonitrile. In the same potential region, ditropyl (**6a**) is oxidized to the tropylium ion (**1b**) (Figure 1) [38].

### 1.1. Spectral Studies

**Figure 2:**
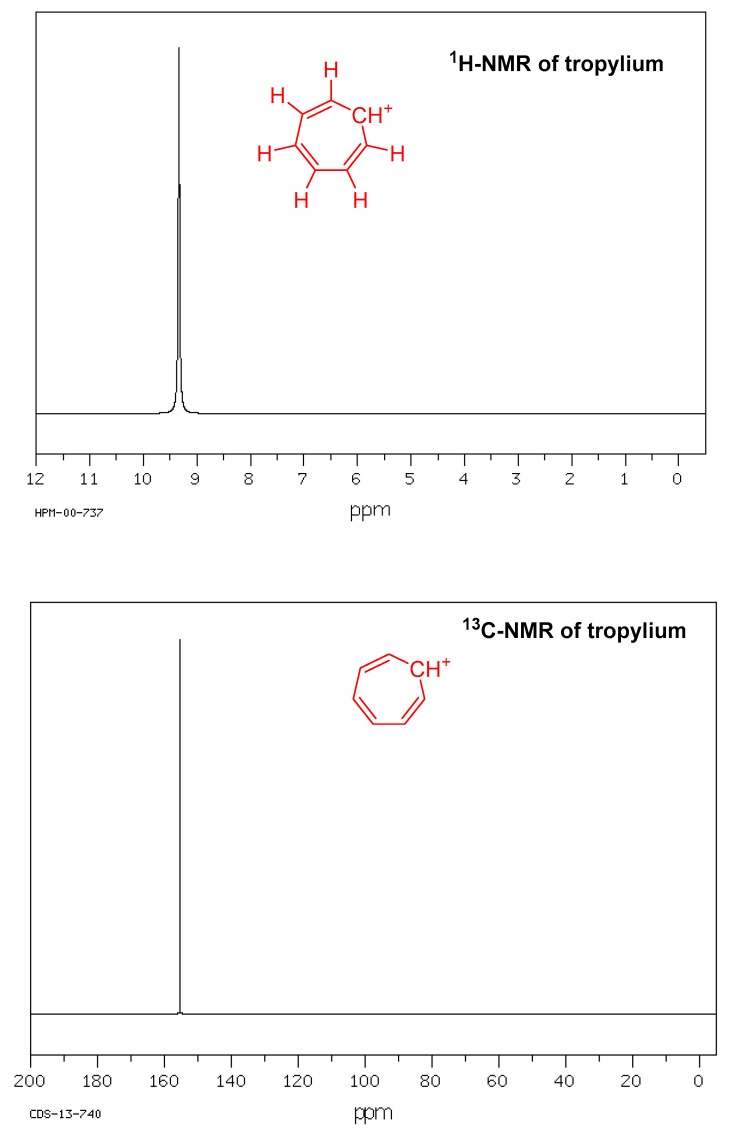
^1^H-NMR and ^13^C-NMR of tropylium ion.

The ^1^H-NMR spectrum of the tropylium ion contains only one peak, showing that all seven protons are equivalent. Similarly, ^13^C-NMR of the tropylium ion also shows one peak, indicating that all seven carbons are equivalent (Figure 2). The ″C, ′H spin–spin coupling constants for tropylium fluoroborate were measured from the ^13^C-NMR spectra of tropylium ions using a distinct experimental technique comprising highly deuterated compounds and ′D-decoupling [39]. The coupling constants for the tropylium ion were found to be ′*J* = 166.79, ′*J*-0, ″*J* = 9.99, and ″*J* = −0.64 Hz [40].

The tropylium ion’s UV spectrum can only be obtained in water at low pH. The specrum is analogous to that of 7-hydroxycycloheptatriene (tropyl alcohol) at high pH and mixtures at intermediate acidity. The tropylium ion has a λmin of 247 nm (log e 3.60) and a λmax of 275 nm (log e 3.64). Only four bands of reasonable intensity could be seen in the infrared spectra of tropylium bromide [41]. Compared to the spectra of the isomeric bromo- and chlorotropilidenes, which are more complex and exhibit at least twelve bands of moderate intensity, this simplicity is more compatible with the high symmetry anticipated for the tropylium ion [42].

**Figure 3:**
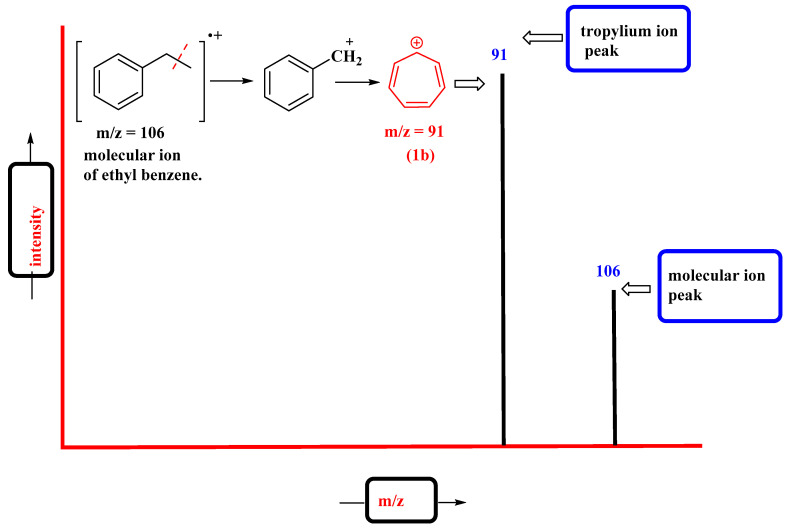
Mass spectrum of ethyl benzene representing characteristic tropylium ion peak.

The tropylium cation is a common fragment in the mass spectra of molecules containing benzyl units. The characteristic signal appears at m/z = 91. It represents the common benzyl fragment (PhCH_2_^+^), which, upon rearrangement, forms the highly stable tropylium cation (C_7_H_7_^+^). For example, the molecular ion peak of ethyl benzene appears at m/z = 106, which, upon ionization, undergoes benzylic cleavage to give the tropylium ion base peak at m/z = 91 (Figure 3).

The spectra of [M + H]^+^ ions of 1,3,5-cycloheptatriene and various 7-alkyl-1,3,5- cycloheptatrienes were measured after gas-phase protonation [43]. Ring contraction of the dihydrotropylium ions to protonated toluene (toluenium ions) and protonated ethyl benzene (ethyl benzenium ions) before fragmentation [44] is indicated by the loss of CH_4_ from the parent ion [1 + H]^+^ and the virtually exclusive loss of C_2_H_4_ from the methyl derivative [3 + H]^+^. Ions [3+ H]^+^ isomerize to xylenium ions as protonation becomes more exothermic [45].

According to ^1^H-NMR studies, (**1a**) is 30% as aromatic as benzene, and tropylium is 22–50% as aromatic as benzene [46]. The annelated dihydropyrenes (tropone-fused and 1,3,5-cycloheptatriene-fused) were synthesized to achieve these estimations (tropylium-fused). The tropylium cation was analyzed after treatment with HBF_4_ [47]. The isomeric cycloheptatrienes (70% yield) were produced when the ketone was reduced with AlH_3_ (generated from AlCl_3_/LiAlH_4_) in ether/benzene at 25 °C [48].

### 1.2. Reactivity of Tropylium Ion

**Figure 4:**
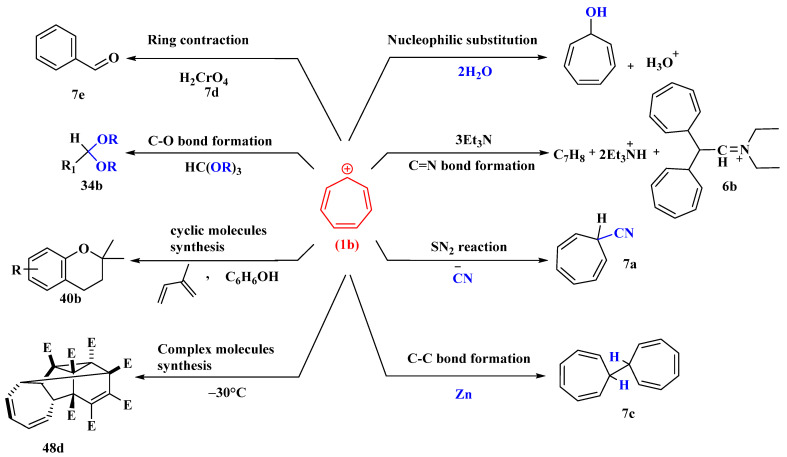
Important reactions of tropylium ion.

The tropylium cation acts as a Lewis acid in the base, water, and is in equilibrium with the covalently bonded carbinol and the hydronium ion (Figure 4). When water is used as the reference base, the tropylium ion has a value of K = 1.8 × 10^−5^, which implies that it is nearly as acidic as acetic acid.

To make substituted cycloheptatrienes, the tropylium cation was treated with the hindered bases triphenylmethyl sodium, 2,6-dimethoxyphenyl lithium, and 2,4,6-tri-*t*-butyl phenyl lithium. Where feasible, it reacts with tertiary amines to produce ammonium salts, which can then be hydrolyzed to form tropylated aldehydes and ketones (Figure 4). When it reacts with trimethylamine, it yields a quaternary ammonium salt, which decomposes into trimethylammonium fluoroborate and a mixture of tropone and ditropyl ether when exposed to the air [49].

The tropylium ion, which can be easily prepared, reacts as an electrophilic reagent with bases such as pyridine (C_5_H_5_N), triethylamine (NEt_3_), and dimethyl sulfoxide (DMSO) to form adducts; with water, hydrogen sulfide, and ammonia to give ditropyl ether, sulfide, and amine, respectively; with the bases acetamide, benzamide, and succinamide to form the *N*-tropyl derivatives; and with cyanide ions to give tropyl cyanide (**7a**), which can be hydrolyzed to an amide (**7b**) and reacts with phenyl magnesium bromide to give desoxybenzoin. The tropylium ion is reduced by zinc dust to ditropyl (**7c**) [50].

Tropyl alcohol is generated first by the interaction of tropylium ions with water, and since it is in equilibrium with the tropylium ions, it reacts further to give the less soluble ether. Ditropyl sulfide is formed when hydrogen sulfide and aqueous tropylium bromide react rapidly. Tropylium bromide reacts similarly to saturated aqueous ammonia to produce ditropylamine, a secondary amine. The primary result of its reaction with ethereal ammonia is tritropylamine, a tertiary amine. Dimethylamine normally reacts with tropylium bromide to form dimethyltropylamine. Tropylium bromide interacts smoothly with aqueous potassium cyanide, giving a liquid product, C_7_H_7_N, in excellent yield. The tropylium ion can be oxidized either by chromic acid (**7d**) in acetic acid or by silver oxide in water to afford benzaldehyde (**7e**) in good yield [51] (Figure 4).

**Figure 5:**
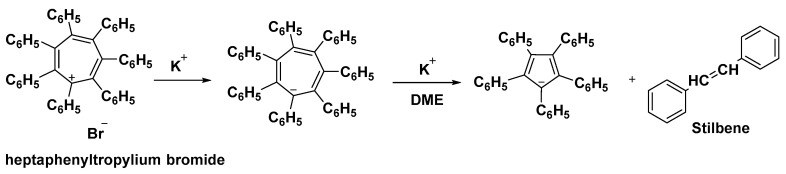
Reduction of heptaphenyl tropylium bromide in presence of potassium ion.

Potassium heptaphenylcycloheptatrienide is formed by reducing heptaphenyltropylium bromide with potassium (Figure 5). The anion reacts with the tropylium ion to form the heptaphenylcycloheptatrienyl radical, whereas reduction by potassium fragments the anion into stilbene and the pentaphenylcyclopentadienyl anion [52].

Sargent and his colleagues discovered that the 7-cycloheptatrienyl group, presumably through its nor-cardienyl valence tautomer, is incredibly effective at stabilizing adjacent cationic carbon atoms. Diazotization of the amine under various circumstances produced the most profound results. Two organic compounds were produced when treated with an equivalent of nitrosonium fluoroborate in acetonitrile at room temperature. Heptafulvene was shown to be the most important [53].

**Figure 6:**
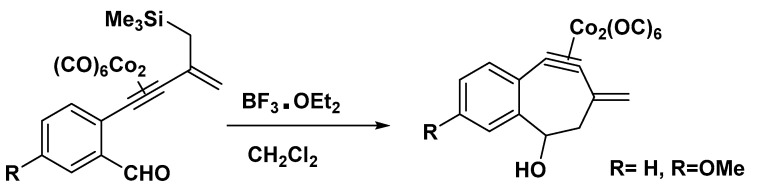
Preparation of a dehydrotropylium ion complex.

It was reported that the generation, trapping, and evaluation of a dehydrotropylium ion resulted in a reduced level of aromatic stabilization. The Jones group produced η^2^-platinum (0), palladium(0), and zirconium(II) complexes of a dehydrotropylium ion or similar cations that exhibit no to moderate C−C bond alternation (Figure 6) [54].

**Figure 7:**
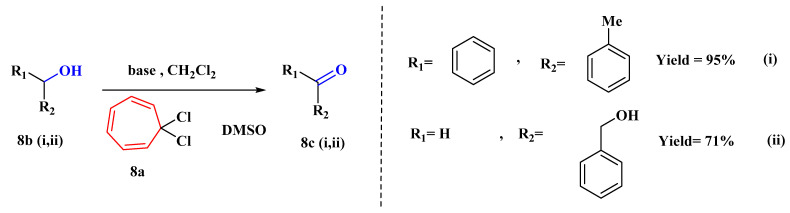
Activation of DMSO using 1,1-dichlorocycloheptatriene.

For a moderate Swern-type oxidation of a range of alcohols (**8b**), a novel dimethyl sulfoxide activation technique using 1,1-dichlorocycloheptatriene (**8a**) has been devised (Figure 7). With this simple and efficient technique, moderate to excellent yields of carbonyl compounds (**8c**) may be produced [55].

In the presence of palladium, the hydrogenation of heptamethyl cycloheptatrieneheptacarboxylate affords a stereoisomer of heptamethyl cyclohepteneheptacarboxylate with the cis position of five ester groups with a yield of 60%, as well as the corresponding cyclohepta-1,3-dienes as trans, trans and trans, cis isomers. After a reduction of one double bond and intramolecular cyclization, the reaction with sodium borohydride resulted in the stereoselective formation of the bicyclic molecule. The chemicals produced were analyzed using X-ray diffraction [56].

**Figure 8:**
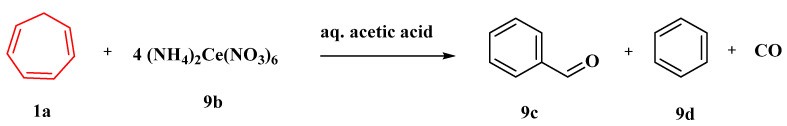
Oxidation of cycloheptatriene by ceric ammonium nitrate.

In aqueous acetic acid and other solvents, the oxidation of 1,3,5-cycloheptatriene (**1a**) by 4 equivalents of ceric ammonium nitrate (**9b**) produces benzaldehyde (**9c**), benzene (**9d**), and carbon monoxide (Figure 8). Three sets of experiments are given, all of which strongly suggest that the tropylium ion is an intermediate in this oxidation. As per the evidence given, the oxygen of the benzaldehyde generated by the ceric ammonium nitrate oxidation of (**1a**) in anhydrous acetonitrile originates from the nitrate ion [57].

**Figure 9:**
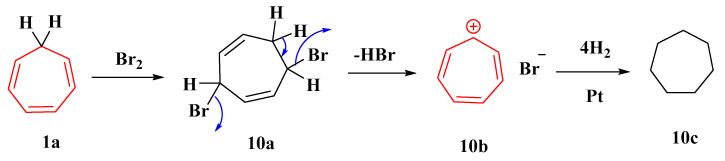
Elimination of HBr from dibromotropilidine.

Dichlorocarbene preferentially adds in a 1,2-fashion to the formal “anti-Bredt”-type double bond of the aromatic ring to produce the nor-caradiene, which immediately rearranges to the bridged cycloheptatriene and then to the isomeric product through a [1,5] sigmatropic chlorine migration [58]. The thermal elimination of hydrogen bromide from dibromotropilidene (**10a**) produces cycloheptatrienylium bromide (**10b**), which is hydrogenated to cycloheptane (**10c**). Tropylium chloride, which is considerably more deliquescent than the bromide, is produced by dissolving tropylium bromide in ethanol, passing it through hydrogen chloride, and precipitating it with ether (Figure 9) [59].

This review aims to provide an overview of the recent developments in tropylium-ion-promoted chemistry, specifically focusing on the tropylium cation (**1b**). This article aims to highlight the recent advances in this emerging field and discuss the potential future directions for research in this area.

## 2. Nucleophilic Substitution Reactions

**Figure 10:**
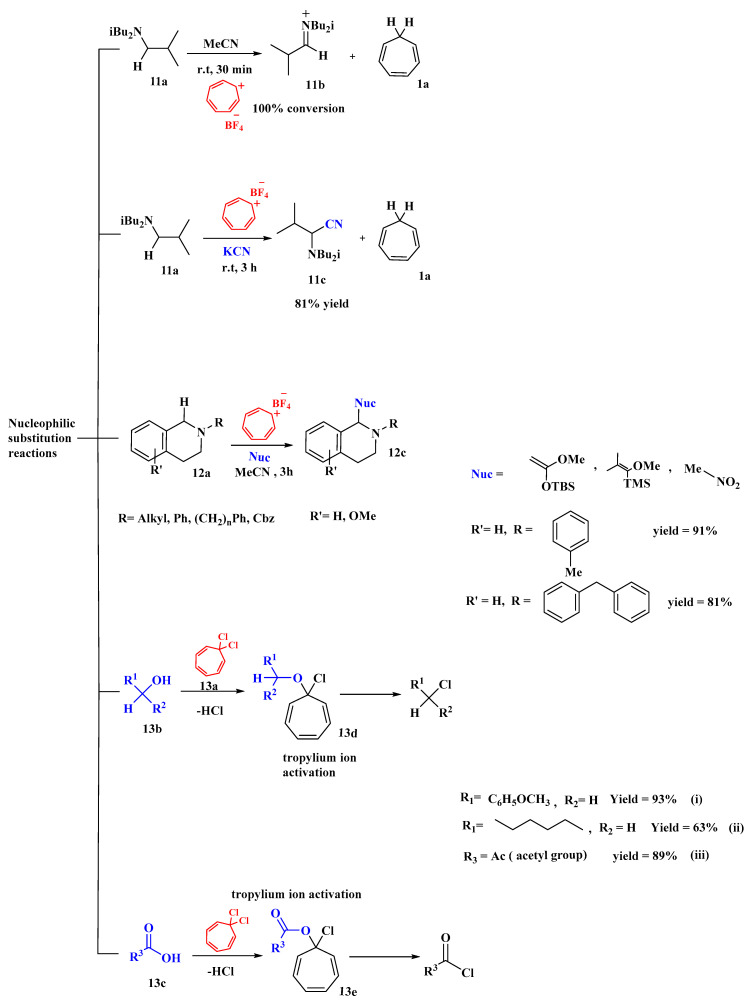
Tropylium as catalyst accelerated nucleophilic substitution reactions.

The oxidizing potential of the tropylium ion to bring about the alpha-cyanation of amines was reported by Lambert [60]. Potassium cyanide (KCN) was used to synthesize aminonitriles, with tropylium being used as a catalyst, as shown in Figure 10. Thirteen substrates were reported, including 17-β-cyanosparteine synthesis (gram scale) and oxidative Aza-Cope rearrangement. The action of the tropylium ion on an amine substrate was investigated. The synthesis of the iminium ion (**11b**) was facilitated in the absence of KCN, but with KCN, it allows the tropylium ion (**1c**) to oxidize the amine substrate (**11a**) and produce alpha-cyanated product (**11c**) in 81% yield. It has a wide range and exhibits regioselective affinity. A high yield (90%) of α-cyanate-sparteine was also found. Substrates containing solely benzylic hydrogens or aliphatic positions that are not reactive caused complications [60].

Nguyen created a new robust method for the oxidative functionalization of tetrahydroiso-quinolines (THIQs) at the C_1_ position using tropylium salts, which has a wide range of applications in medicinal chemistry [61]. He worked on *N*-substituted THIQs with multiple nucleophiles, such as Grignard and nitromethane, and synthesized alkylated and arylated THIQs with solid results through iminium intermediates. These iminium intermediates were generated with silyl enol ethers, which gave quick access to THIQs in good to high yields (**12c**). Furthermore, the alkylation of substrates (**12a**) with nitromethane (**i**) gave products in good yields (**12c**). These tropylium-based processes involved hydride abstraction, accompanied by the nucleophilic quenching of intermediates, and generated cycloheptatriene as a byproduct [61].

A technique for the activation of alcohols using tropylium cations for dehydrative nucleophilic chlorination and bromination was reported by Nguyen et al. [62]. In addition to the activation of alcohols, the researchers proposed that the tropylium cation could also be used to activate carboxylic acids for nucleophilic acyl substitution in the same manner. The reagent 1,1-dichlorocycloheptatriene (**13a**) can be generated in situ or assembled from readily available starting materials and stored under inert conditions for an extended period. The reaction of (**13a**) with alcohols (**13b**) and acid substrates (**13c**) converted them to chlorides and acid chlorides, respectively. The reactivity decreased from an activated primary alcohol to a non-activated primary alcohol and then to a non-activated secondary alcohol. The reaction proceeded via the formation of tropylium alkoxy (**13d**) and (**13e**) and electron-donating and electron-withdrawing groups did not affect the reactivity. The conversion of carboxylic acids (**13e**) to acid chlorides resulted in high yields, but non-activated primary and secondary alcohols (**13b**) required elevated conditions to work and resulted in lower yields (Figure 10). Moreover, it was found that this method of conversion was easy, rapid, and clean, proceeded under mild conditions, and required a short reaction time [62].

## 3. C-N Bond Formation Reactions

**Figure 11:**
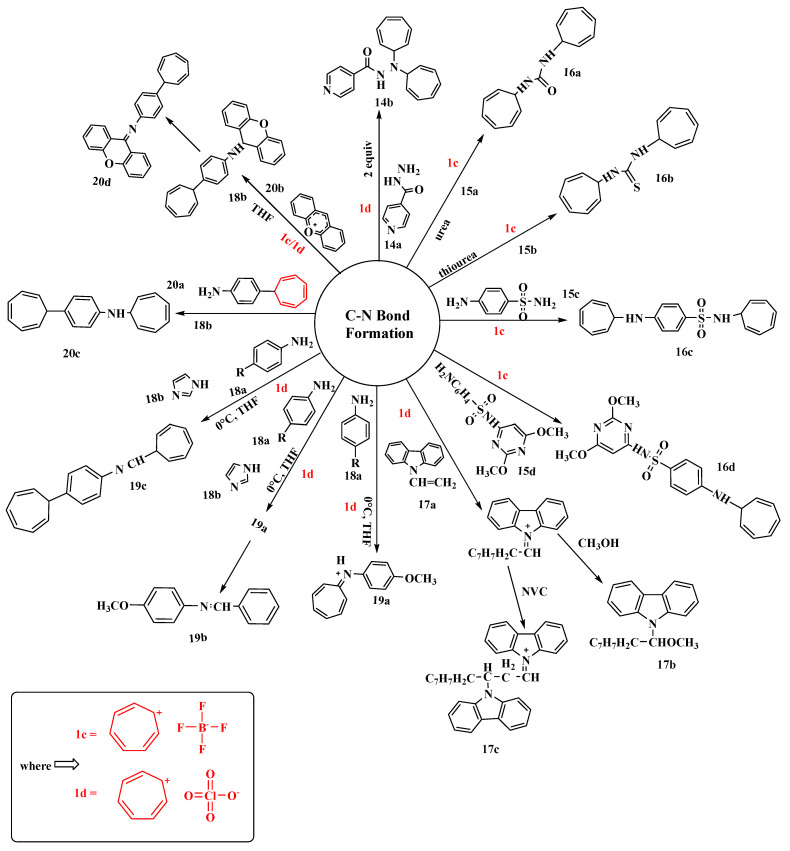
Tropylium-mediated synthesis of compounds containing C-N bonds.

Research based on the reaction of monocarboxylic acid hydrazides (**14a**) with tropylium salts was performed by the Yunnikova research group [63]. They combined three distinct substrates (nicotinic acid hydrazide, isonicotinic acid hydrazide, and furan-2-carbohydrazide) with two different tropylium salts, perchlorate (**1d**) and tetrafluoroborate (**1c**), to create biologically significant products. The author’s work was praised since the technique was straightforward, the reactions were carried out in water at room temperature, and good yields were obtained. Using tropylium perchlorate (**1d**), the product *N*′*, N*′-di(cyclohepta-2,4,6-trien-1-yl)pyridine-4-carbohydrazide (**14b**) was produced with the highest yield (93%) (Figure 11). The products were further clarified based on NMR and mass spectrometry (MS) studies [63].

By reacting urea (**15a**), thiourea (**15b**), sulfonamide (**15c**), or sulfadimethoxine (**15d**) with (**1c**), novel compounds were produced by substituting a hydrogen atom with tropylium at the amide nitrogen or the amino group of the benzene ring. Furthermore, the tropylium-salt-containing 1,3,5-cycloheptatriene was quite stable and possessed unique pharmacological properties. The primary goal of this research was to apply tropylation reactions to the graph of a well-known antimicrobial compound. Compounds such as *N,N*′-di(cyclohepta-2,4,6-trien-1yl)urea (**16a**), *N,N*′-di(cyclohepta-2,4,6-trien-1-yl)thiourea (**16b**), *N-*(cyclohepta-2,4,6-trien-1-yl)-4-(cyclohepta-2′,4′,6′-trien-1-yl-amino)benzenesulfonamide (**16c**), and sulfadimethoxine [4-amino-*N*-(2,6-dimethoxy-4-pyrimidinyl)benzenesulfonamide] (**16d**) have been synthesized [64].

Bowyer, along with his colleagues, reported the cationic polymerization of *N*-vinyl carbazole (**17a**) (NVC) initiated by tropylium hexachloroantimonate and tropylium perchlorate (**1d**) (Figure 11) [65]. The adiabatic calorimetric technique was used to determine the rates of reaction, and the order of the half-life was 1–2 s. The initiation reaction of the tropylium ion with a monomer occurs readily with ion pairs (low concentration), whereas propagation occurs through free cations. To start the process, stable carbonium ion salts were employed. It was observed that the polymer (**17c**) was formed in high yield through a charge-transfer complex when the two reagents (**17a**) and (**1d**) reacted at a temperature above −50 degrees. The molecular weight of the polymer produced using perchlorate catalysis was found to be lower than that of the polymer created via hexachloroantimonate catalysis. Conductance measurements were utilized to investigate the tropylium salt ion-pair dissociation equilibria [65].

The tropylation of arylamines using tropylium perchlorate (**1d**) was studied by Yunnikova [66]. She was able to synthesize 4-(7-cyclohepta-1,3,5-trienyl)-*N*-(1-cyclohepta-2,4,6-trienyl)aniline by the tropylation of *p*-anisidine with (**1d**), which formed the salt 8-*p*-methoxyphenyl-8-azaheptafulvenium perchlorate (**19a**) in 72% yield. Because of the tropylium ring contraction in the presence of imidazole, this product was transformed into *N*-benzylidene-4-methoxyaniline (**19b**). The yield of (**19b**) was a bit higher (76%) than that of (**19a**) due to the presence of imidazole (**18b**) as an activator. Moreover, replacing the methoxy in *p*-anisidine with tropylium altered the reaction’s path and produced (**19c**) in a yield 56% less than the previous compounds synthesized [66].

Yunnikova’s research group proposed that tropylium, xanthylium, and tritylium salts have various stabilities, which affect how they react with biologically active amines [67]. The reaction of (**1d**) and (**1c**) with 4-(cyclohepta-2,4,6-trien-1-yl) aniline (**20a**), followed by the hydrolysis of the *N*-(cyclohepta-2,4,6-trien-1-yl) derivative, is responsible for the difference in their relativities. Tritylium perchlorate failed to react with (**20a**), whereas the *N*-xanthenyl derivative was dehydrogenated. The reactions of pyrimidin-2-amine with tropylium, xanthylium, and tritylium salts produced large yields with the replacement of one hydrogen atom in the amino group (Figure 8). In the synthesis of (**20c**), the tropylium ring remained intact with a good yield, but when using two equivalents of (**20b**), the yield increased from 44% to 87%. The purpose of this work was to investigate amine reactions with tropylium, xanthylium, and tritylium salts and estimate the stability and yields of the products. ^1^H-NMR and mass spectra, as well as the X-ray analysis of amines, were used to confirm the structure of all isolated compounds [67].

## 4. C-C Bond Formation Reactions

**Figure 12:**
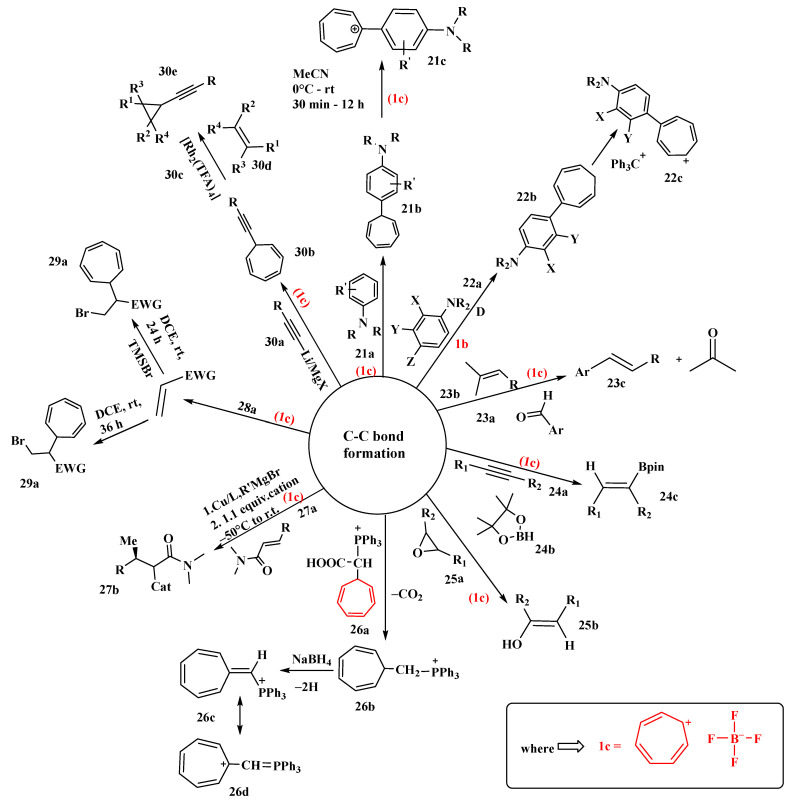
Tropylium-promoted synthesis containing C-C bond formation.

Nguyen’s research group successfully created a series of new cationic organic dyes from widely accessible synthetic precursors utilizing the tropylium ion as an electron-withdrawing chromophore in a simple one-pot chemical method [68]. The synthesis was carried out by mixing aniline (**21a**) and two equivalents of (**1c**) to produce (**21b**) and (**21c**) in good to excellent yields. The reaction proceeded with electrophilic aromatic substitution with the first equivalent of (**1c**) and by hydride abstraction with the second equivalent. By controlling the reaction conditions, the intermediate (**21b**) could be isolated (Figure 12). These tropylium-based organic dyes were stable and water-soluble, with high visual absorption, as demonstrated by easy and practical synthesis methods. All of the organic dyes mentioned in this article were pH-responsive and exhibited unique physicochemical properties, such as solvatochromism, redox-responsivity, Lewis basicity, and fluoride anion sensitivity; however, the researchers only looked at two members of this family, Trop-26DIPA (diisopropylamine) and Trop-DEA (diethanolamine) [68].

Looker proposed the use of tropylium tetrafluoroborate (**1c**) in the alkylation of aromatic rings such as 1-dimethylaminonaphthalene, *N, N*-dimethylaniline, and *N, N*-diethyl-m-toluidine (**22a**), activated by dialkylamino groups [69]. NMR studies revealed two sets of peaks, indicating that the products obtained were isomeric mixtures of dialkylaminoarylcycloheptatrienes (**22b**) formed via double-bond shifts in seven-membered rings. Some of these isomers were isolated in yields of 80–84%. The thermal isomerization of 7-substituted 1,3,5-cycloheptatriene was examined by NMR studies. Triphenylmethyl fluoroborate was used to oxidize the substituted cycloheptatriene to the corresponding tropylium salt alkylating agent (**22c**), generating deep-blue, stable compounds (Figure 12) [69].

Based on preliminary studies of olefin–olefin metathesis reactions, the Nguyen research group investigated the carbonyl olefin metathesis reaction [70]. In these reactions, they illustrated the use of tropylium as an organocatalyst. Tropylium catalysts were used in intramolecular, intermolecular, and ring-opening carbonyl olefin metathesis (COM) reactions in their research. They started their investigation with intramolecular COM reactions using different aldehydes (**23a**) and isopropylidine-bearing olefin substrates (**23b**). The final product formed was a trans-isomer (**23c**) with a good yield. It was further proved that electron-deficient aryl aldehydes did not lead to any productive outcomes. Tropylium ions preferred both inter- and intramolecular interactions, but with intramolecular reactions, a 93% yield was achieved. Catalysts such as iron chloride and tritylium ions, which were previously employed, favored only one type [70].

A specific method for efficiently promoting the hydroboration reaction of alkynes (**24a**) and epoxides (**25a**) by utilizing tropylium salts, which are often used as organic oxidants and Lewis acids, is described. The tropylium ion (**1b**) may abstract a hydride from borane reagents (**24b**) and use it to activate the reaction by forming a borenium ion. The catalytic activity of (**1c**) in the synthesis of phenylacetylene (**25b**) and pinacolborane (**24c**) was studied. The fact that potassium or tetrabutylammonium salts of the same counterion either had no catalytic activity or hindered the reaction emphasized tropylium’s importance. It is noteworthy to mention the authors whose study indicated that tropylium salts function as efficient reaction promoters and that hydride abstraction of pinacol borane with the tropylium ion initiated several intriguing pathways of reactions. A variety of substrates were tested, and it was shown that a satisfactory yield could be obtained with a 5% mole tropylium catalyst and a 12 h reaction time. However, with epoxide substrates (**25a**), a high catalytic amount and a longer reaction time are needed to give the completely hydrolyzed product (**25b**) [71].

Triphenylphosphonium tetrafluoroborates were synthesized by Cavicchio et al. [72]. They examined the possibility of synthesizing onium salts and ylides due to the possible synthetic utility in the realm of cycloheptatriene and tropylium ion derivatives. They discovered in preliminary research that the simple direct route to phosphonium salts of (**26a**) through the interaction of the tropylium ion (**1b**) with phosphonium ylides is confined to stabilized ylides from (**26c**) but fails when R = H (**26b**) due to the extremely high reactivity of methylenetriphenylphosphorane (Figure 12). The authors were successful in explaining the stabilizing effect of the phosphonium group on the heptafulvene system, comparable to cyano or carbonyl groups. The synthesis of (**26c**) was reported, which was then successfully converted to (**26d**) [72].

Sebesta studied different techniques for trapping chiral enolates (ideally silyl enolates) generated by Lewis acids, which are synthesized by the enantioselective conjugate addition of Grignard reagents to unreactive Michael acceptors [73] (Figure 12). This one-pot reaction was accomplished by the use of different electrophilic reagents. The trapping of Mg-enolates by carbocations was reported. The tropylium cation was the first electrophile to be utilized. The investigation started with amides (**27a**) that led to functionalized molecules (**27b**). The yield was determined by changing the solvents, reagent side chains, and electrophiles. The polar additive 1,3-dimethylimidazolidin-2-one (DMEU) was added, as it afforded the highest conversion. It was discovered experimentally that steric parameters play a significant role in the reaction outcome and that trapping reactions are highly dependent on the electrophile structure. A rapid decrease in the yield was observed as the R group became bulkier (**27a**). The overall yields of all trapping reactions varied from 15 to 65%, and it was assumed that the low yields were due to silyl enolate’s poor reactivity. It is worth mentioning the author, whose study revealed that numerous functionalized products may be produced using a single-pot approach that was previously unattainable using other methods [73].

Nguyen developed a new method using halide ions for activating electron-deficient olefins for nucleophilic attack on stabilized carbenium ions in a catalyst-free, metal-free, non-radical fashion [74]. This new chemical reaction thus allows the direct carbohalogenation of electron-deficient olefins. He discovered that tropylium bromide interacted with electron-deficient alkenes (**28a**) such as methyl acrylate in a highly regioselective manner to form the racemic bromocyclohepta-trienylated compound (**29a**). The substrates with C-C multiple bonds without acceptor substituents such as amide groups gave high yields up to 91%. He also described transformations that allowed easy access to a diverse variety of synthetically valuable substituted cycloheptatrienes. The author further revealed that electron-deficient alkynes behaved similarly to their olefinic analogs [74].

A two-step alkynylcarbene transfer reaction for the assembly of alkynyl cyclopropanes was developed using commercially available tropylium tetrafluoroborate, terminal alkynes, and alkenes. The reaction proceeded through the decarbenation of readily available 7-alkynyl cycloheptatrienes (**30b**) (prepared in one step from commercially available terminal alkynes (**30a**) and (**1c**)). This reaction was catalyzed by rhodium (II), which outmaneuvers the fundamental problem associated with 1,6-enyne-containing substrates with Lewis acids, which usually evolve via 6-endo-dig cyclization or ring-contraction pathways under metal catalysis. Based on this discovery, the researchers were able to synthesize a wide variety of cis-alkynylcyclopropanes (**30e**) bearing C (sp^3−^), C (sp^2^)-, and C (sp)-, H-, Si-, or Ge substituents in the alkyne terminus (Figure 12). It was observed that (**30b**) with bulky alkyl groups and phenyl groups with electron-donating substituents led to high yields of (**30e**). Moreover, less activated alkenes and electron-rich alkenes (**30d**) were both compatible with the reaction conditions (Figure 12). The formation of rhodium (II) alkynylcarbene intermediates, which react smoothly with alkenes (**30d**) to give cyclopropanes, was also supported by experimental and theoretical investigations [75].

## 5. Ring-Contraction Reactions

**Figure 13:**
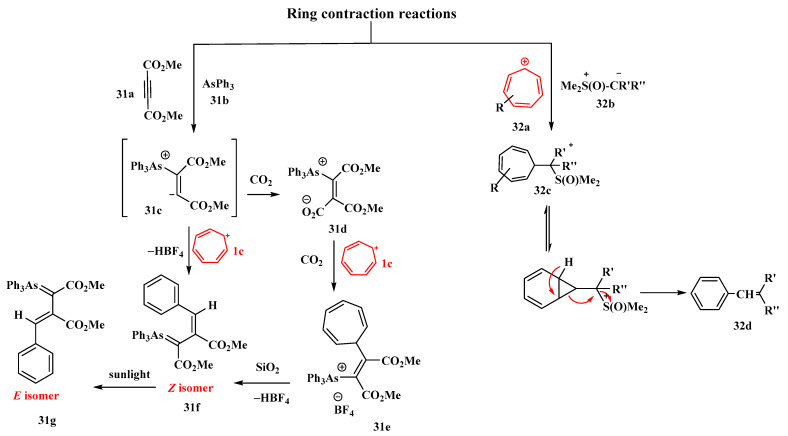
Role of tropylium in ring-contraction reactions.

The relevance of the ring contraction of cycloheptatriene is highlighted by Alsamarrai [76]. The interaction of tropylium fluoroborate (**1c**) and arsonium ylide (**31d**) in dichloromethane produced 3-phenyl-1,2-dicarbomethoxyprop-2-enylidentriphenyl arsonium salt (**31e**). The arsonium ylide was synthesized by the reaction of (**31b**) and (**31a**) dimethyl acetylenedicarboxylate (DMAD). The product (**31e**) underwent further treatment with aqueous NaOH to obtain the *Z*-isomer arsorane (**31f**) (a colorless gum, yield = 77.7%) (Figure 13). It involved the cycloheptatriene ring contraction of the salt (**31e**). The *Z-*isomer (**31f**) then isomerized to the *E*-isomer (**31g**) under sunlight irradiation and was extremely important. The relationship between the two isomers was determined to be geometrical isomerism by utilizing analytical HPLC. An alternate approach to the isomeric product (**31f**) was also devised by directly treating (**31c**) with (**1c**) in acetonitrile, which proved to be a more efficient procedure. The reactivity of isomer (**31f**) with (**1c**) was further investigated [76].

Caviccbio, together with his colleagues, explored the reaction of a few ylides and the associated onium salts with the tropylium ion [77] (**32a**) to produce cycloheptatrienyl onium salts (**32c**) in greater depth. They believed that by using the appropriate reaction conditions or leaving groups weaker than dimethylsulfoxide, they might isolate tropenyl onium salts (DMS or DMSO). Based on previous research on the reaction of tropylium ions with stabilized sulfonium and oxosulfonium ylide (**32b**) (Figure 13), they first reacted some carbonyl stabilized phosphorous, arsenic, and sulfur ylides with tropylium salts in tryptophan (TRP) at r.t and discovered that, while the first step constantly leads to the tropenyl salt and the reaction stops, the salt can be isolated when there is a relatively poor leaving group in the ylide moiety, and the salt cannot be isolated when there is a good leaving group. The reaction between the tropylium cation and arsonium ylide exhibited similar characteristics. The final product trans-chalcone (**32d**) was formed from the intermediate (**32c**) in 90% yield. An attempt to obtain the same result by reacting the tropylium ion with dimethylphenacylsulfonium fluoroborate failed. The sulfonium salt was found to be significantly less stable than the related phosphonium and arsonium compounds [77].

## 6. C-O Bond Formation Reactions

**Figure 14:**
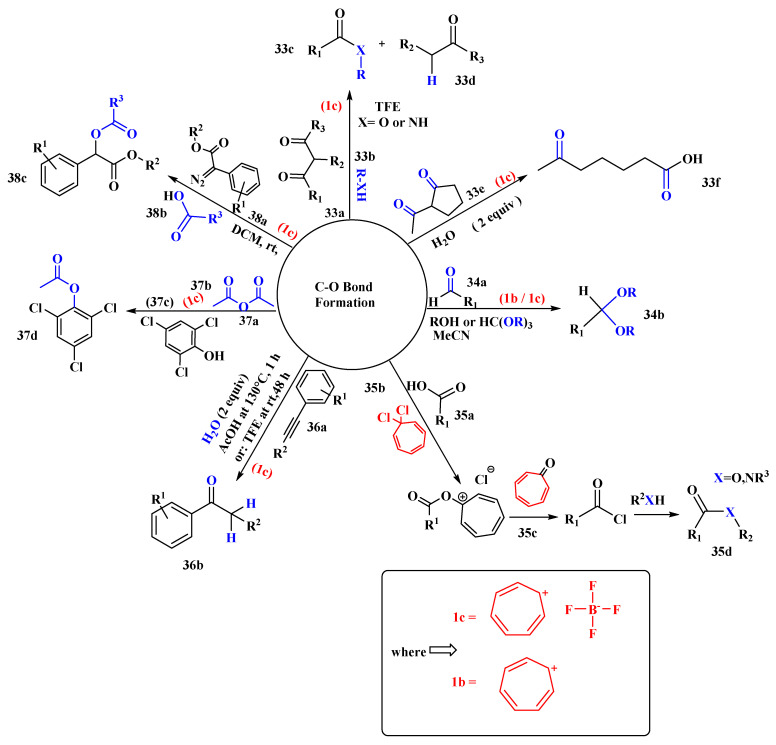
Tropylium-accelerated synthesis of compounds containing C-O bonds.

Nguyen discovered a novel technique for the C-C bond cleavage of 1,3 diketones based on the retro-Claisen reaction and ester derivative synthesis [78]. This work was notable since it utilized the organocatalyst tropylium tetrafluoroborate (**1c**) instead of the previously used transition-metal Lewis acid catalysts and organic Bronsted acids/bases. They utilized this newly developed procedure to perform a range of hydrolysis, alcoholytic, and aminolytic reactions on cyclic diketones (**33a**) (Figure 14). The hydrolysis of 2-acetyl-cyclopentantone (**33e**) of the retro-Claisen type was achieved with a high yield of 99% using the ionizing solvent trifluoroethanol (TFE). The hydrolysis product (**33f**) was obtained by a tropylium-catalyzed dehydration reaction. Using an alternative batch or flow technique, a wide range of carboxylic acid, ester, amide, and thioether products were formed using water, alcohols, amines, and mercaptans [78].

Nguyen et al. demonstrated the use of tropylium ions as Lewis acid catalysts in acetalization and transacetalization reactions of carbonyl compounds with 1,2-diols [79]. Their research relied on a diverse set of aldehyde substrates (**34a**) since ketones were insufficiently reactive with tropylium-catalyzed reactions. They proved that all benzaldehydes (**34a**) reacted smoothly to give diethyl and dimethyl acetal products (**34b**) in excellent yields (99%), but heteroaromatic aldehydes such as (**34a**) were only partially converted to products (9%) and not acetalized at all (Figure 14). Apart from being a metal-free and environmentally friendly approach, it was also beneficial for aldehyde chemoselectivity and was shown to operate successfully in both batch and flow conditions. The authors’ work paved the way for a new approach in the field of green chemistry by employing the tropylium ion as an organocatalyst [79].

A unique method developed by Nguyen for carboxylic acid coupling reactions led to the production of esters, amides, lactones, and peptides in high yields [80]. The research was focused on 1,1-dichlorocycloheptatriene (a new chlorinated coupling regent **35b**) due to its ability to equilibrate between the neutral cycloheptatriene and tropylium ion forms. Both aliphatic and aromatic acids (**35a**) were utilized, but the latter reacted under sluggish conditions due to decreased nucleophilicity. Low to high yields were reported with various alcohols, such as phenols with varied substituents. It was demonstrated that electron-donating groups enhanced the yield (**35d**), whereas electron-withdrawing groups lowered the yield. The optimal conditions for this reaction required dichloromethane as a solvent in the presence of an organic base. The intriguing aspect was the byproduct tropone (**35c**), which might be used to rejuvenate the (**35b**) coupling reagent [80].

Nguyen introduced a novel strategy for adding water across an alkyne’s carbon–carbon triple bond, producing a ketone [81]. Their experiments centered on the use of the tropylium ion (**1c**) as an organocatalyst instead of metal catalysts (Figure 14). A variety of alkyne substrates and solvents were explored, and the yield was evaluated. The solvents tetrafluoroethylene (TFE) and acetic acid (AcOH) were used to achieve a 100% yield. It was observed that all electron-neutral and electron-donating substituents (**36a**) reacted smoothly to give the corresponding acetophenones (**36b**), but strong electron-withdrawing groups such as nitro (**36a**) could not be hydrated. This proved that an π-electron-rich system is required for tropylium activation. Moreover, phenyl acetylenes with halogen substituents (**36a**) seemed to be less reactive and required harsh conditions to be hydrated. This method gave good yields and paved the path for future research in synthetic chemistry, but it was confined to only electron-rich aromatic alkynes [81].

Tropolonate salts act as acyl transfer catalysts under thermal and photochemical conditions, as shown by Nguyen et al. They developed a novel acyl transfer paradigm based on the tropolone scaffold, which may strongly enhance the coupling reaction between alcohols and carboxylic acid anhydrides or chlorides to yield ester compounds (**37d**). The authors also stated that tropolonate is a suitable leaving group for the acyl transfer reaction. The studies used phenol 2, 4, 6-trichlorophenol (**37a**) as the model substrate in a reaction with acetic anhydride (**37b**), with tropolonate salts (**37c**) acting as reaction promoters (Figure 14). For the process, a variety of frequently used organic solvents were examined, and acetonitrile appeared to be ideal, presumably with the best tropolonate solubility, while potassium tropolonate provided the highest efficiency. The acylation reactions of a variety of alcohols and phenols with pivaloyl and benzoyl chlorides produced high to excellent yields [82].

The novel catalytic activity of the tropylium ion to enhance OH functionalization reactions of aryldiazoacetates with carboxylic acids is highlighted in this paper. This new method, developed by Koenigs et al. [83], applies to a wide range of diazoalkane and carboxylic acid derivatives, including aromatic, heteroaromatic, and aliphatic carboxylic acids, to give α-functionalized esters (**38c**) with excellent efficiency up to a 99% yield. The researchers started their investigation by looking into tropylium (**1c**)-promoted diazoalkane O−H functionalization (**38a**) with benzoic acids (**38b**) and extended their investigation to different diazoalkanes. In the case of linear alkyl esters (**38a**), a high yield was observed, but a yield drop was visible with bulky groups. They further found that all of the alkyl carboxylic acids worked smoothly to give the desired O−H functionalization products in good to high yields. With unsubstituted benzoic acid (**38b**), a 97% yield of (**38c**) was observed, but it decreased with bulky group substitution (**38b**) (Figure 14). The reaction is simple and proceeds under metal-free mild reaction conditions with very short reaction times [83].

## 7. Cyclic Molecule Synthesis

**Figure 15:**
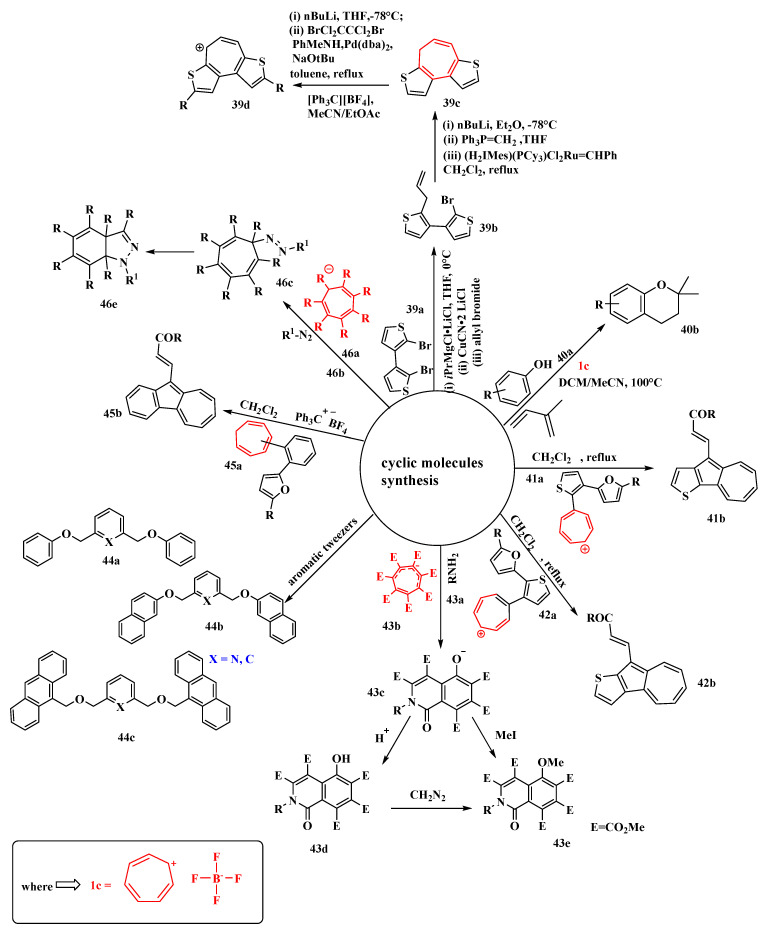
Tropylium-promoted synthesis of cyclic compounds.

The synthesis of extremely stable thiophene-based tropylium cationic dyes was reported by the Fukazawa research group [84]. Thiophene-fused tropylium cations with terminal amino groups displayed exceptional stability (because of aromaticity) and high pka values. A quinoidal character was discovered during X-ray crystallographic analysis owing to electron-donating amino groups that decrease the aromaticity of the thiophene rings and tropylium cation. Moreover, the introduction of electron-donating groups on the fifth position of the bithiophene ring (**39a**) contributed to two resonance structures of (**39d**), which exhibited intense absorption and fluorescence properties. A series of amino-modified dithienotropylium ions were produced utilizing 4*H*-cycloheptal [1,2-β;4,3-β′] dithiophene (**39c**) as a key intermediate (Figure 15). Orange to red fluorescence was observed due to absorption in the visible region. This photophysical property relied on the 3, 3′-bithiophene-fused substructure [84].

Nguyen and his colleagues proposed a novel method for synthesizing 2,2-dimethylchromans, a biologically active and significant medicinal substance [85]. The use of tropylium (**1c**) as a Lewis acid catalyst in the prenylation reaction for the synthesis of 2,2-dimethylchromans (**40b**) was the key discovery since the catalyst was found to efficiently induce these reactions. The technique was examined by modifying the reaction conditions and utilizing different solvents, such as dichloromethane (DCM), methyl cyanide (MeCN), and 1,2-dichloroethane (DCE). The maximum yield (89%) was achieved at high temperatures using DCM as the solvent (Figure 15). It was also discovered that electron-rich phenols, such as (**40a**), provided rapid access to the product (**40b**) in excellent yields of up to 95%, but electron-deficient phenols, such as *p*-halide phenols (**40a**), were not converted to products and remained in the reaction mixture. Overall, the approach was less time-consuming, less costly, metal-free, and based on plug-flow chemistry, which resolved the problem of side polymerization products but could not function with electron-deficient phenols [85].

Yamamura proposed an innovative way of synthesizing benz[*α*]azulenic enones [86] based on the salt-promoted intramolecular reaction of *o*-(2-furyl)-cycloheptatrienyl benzenes (**41a** and **42a**). This is the first example of tropylium-ion-mediated furan-ring-opening reactions that result in benz[*α*]azulene rings (**41b** and **42b**). ^1^H-NMR and mass spectra, as well as elemental analyses, verified the structures of the obtained β-(4-azuleno [1,2-β]thienyl)-α,β-unsaturated ketones (**41b**) and β-(4-azuleno [2,1-β]thienyl)-α,β-unsaturated ketones (**42b**). The valuable work of the author revealed an efficient synthetic method for the synthesis of (**41b** and **42b**) by refluxing dichloromethane solutions of 2-tropylio-3-(2-furyl)-thiophene tetrafluoroborate (**41a**) and 3-tropylio-2-(2-furyl)-thiophene tetrafluoroborates (**42a**), respectively, and moderate yields were obtained (Figure 15). The alkyl, alkenyl, and phenyl substituents on the furan ring were successfully converted to the corresponding azulenic enones, except for the formyl substituent, for which the conversion was not observed [86].

The action of bases on propenyl pyridinones induces cyclization into 1-oxodihydroisoquinolin-5-olates (**43c**), which, upon acid treatment, generates 5-hydroxyhydroisoquinolin-1-ones (**43d**) in high yields. This method was demonstrated by Tomilov and his coworkers [87]. The rate of cyclization is determined by the base strength. The reaction takes only a few minutes with strong bases such as KOH, while it takes 10–20 h with primary amines. Moreover, the author also studied the direct reaction of heptamethoxycarbonylcyclohepta-2,4,6-trien-1-yl potassium with cyclopropylamine. The reaction of cyclopropylamine with cycloheptatrienyl potassium (**43b**) in acetonitrile solution at 5 °C for 16 h led to the formation of potassium dihydroisoquinolin-5-olate (**43c**) in 85% yield (Figure 15) [87].

The characterization of novel aromatic tweezers and complex formation with the tropylium ion (**1b**) in 1,2-dichloroethane was discussed by the Lamsa research group [88]. π-π stacking interactions were found to be crucial in the complexation of benzene-substituted crown ethers with certain electron-deficient ions, such as tropylium pyridinium. It is important to note the author, whose work indicated that complexation studies of crown ethers and podands with organic cations may be used as a starting point for the creation of tweezer structures. In the presence of the tropylium ion, the electronic spectra of the tweezers exhibited a broad wavelength absorption at 300–600 nm. The structures (**44a**) and (**44b**) were synthesized simply by refluxing the reactants with potassium carbonate and acetone as a solvent. The tweezers (**44c**) were prepared by using sodium hydride in dry DMF with 39% to 78% yields and exhibited properties of complexation and intramolecular cyclization [88].

Yamamura, along with his coworkers, reported a facile, one-pot synthesis of β-(benz[*a*]azulen-10-yl)-α,β-unsaturated ketones from the corresponding *o*-(2-furyl)cyclohepta- trienyl benzenes (**45a**) [89]. The mechanism involves a unique ring-opening cyclization reaction via the intramolecular attack of the tropylium ion on the 2-position of the furan ring. The common starting material for the synthesis of (**45b**), *o*-cycloheptatrienylbromobenzene, was prepared as an isomeric mixture of commercially available cyclohepta-1,3,5-triene and *o*-bromoiodobenzene by utilizing Heck arylation. Proceeding via the Stille coupling reaction, (**45a**) were synthesized, which were then further converted to benz[*a*]azulenic enones (**45b**) (Figure 15). The yield of (**45b**) was in the range of 32 to 48%, except when (R = H), where the yield was reduced to 25%, which was improved to 44% by using 2 equivalents of the salt in this case. This technique emphasizes the significance of the reaction since it is simple to use, and (**45b**) is difficult to synthesize by other methods [89].

Tomilov et al. analyzed and detailed the synthesis of heptaester-substituted cycloheptatriene using a one-pot technique [90]. They accomplished this by starting with simple materials, such as one molecule of methyl diazoacetate and three molecules of 2, 3-dibromosuccinate in pyridine. The base reaction produced the cycloheptatrienyl anion (**46a**), which they isolated and investigated in conjunction with electrophiles such as methyl iodide, allyl bromide, and diazonium salts (**46b**). The azo coupling reaction was performed between (**36a**) and (**46b**), and the expected product was (**46c**), but NMR results confirmed the formation of dihydroindazole derivatives (**46e**) in high yields up to 80%. The product (**46e**) was possible due to the ring contraction of the intermediate (**46c**) (Figure 15). With the latter, they were able to isolate derivatives of dihydroindazole in conditions of cesium carbonate and nitrosourea. They also found that the multiple bonds in the side chains of products, when subjected to further transformations, such as intramolecular transformations, resulted in the formation of a cage structure and heterocyclic compounds [90].

## 8. Complex Molecule Synthesis

**Figure 16:**
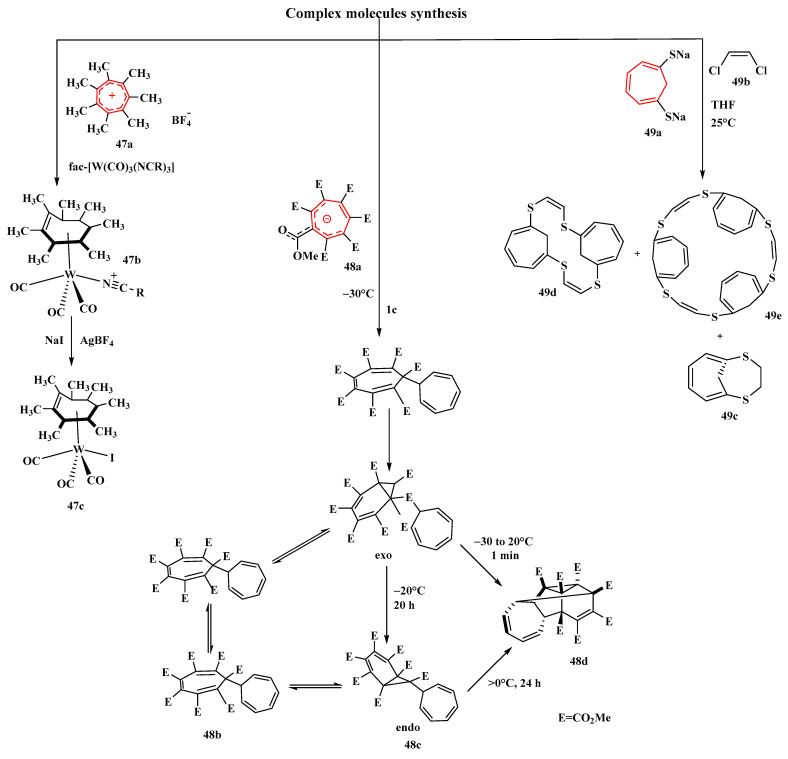
Synthesis of complex compounds promoted by tropylium.

Tamm published an improved protocol for the production of remarkably stable and easy-to-assemble η^5^-heptamethylcycloheptatrienyl complexes (**47c**) [91]. They observed that the coordination of the sterically demanding cation (**47a**) to transition-metal centers was facile, and thus, the researchers were able to successfully prepare the first transition-metal complexes containing this molecular cogwheel. By substituting the three nitrile ligands, tropylium salts can produce cationic cycloheptatrienyl complexes [(η^7^-C_7_R_7_)M(CO)_3_]^+^ (**47b**) by reacting with complexes of the type fac-[M(CO)_3_(NCR)_3_] (M = Mo, W; R = Me, Et). The cationic tungsten complex (**47b**) reacts with halides to form neutrally charged complexes (**47c**) [(η^7^-1,2,4,6-C_7_Me_4_H_3_)W(CO)_2_X] (X = Br, I), which is consistent with the reactivity of the related cationic molybdenum and tungsten tricarbonyl complexes (Figure 16). These structures proved to be promising candidates for further reactions, such as for the preparation of bimetallic complexes [91].

Tomilov and his coworkers reported an unusual aspect of the tropylium cation (**1b**) implicated in the development of cage structures [92]. Their research was based on a multistep reaction involving two oppositely charged ions of seven-membered carbocycles, tropylium cation (**1c**), and the hepta(methoxycarbonyl)cycloheptatriene (HMCH) anion (**48a**), which proceeded via a sandwich-type intermediate. This was then converted to a cage structure (**48d**) and stable isomers (endo **48c**) by intramolecular cyclization and isomerization under varying reaction conditions (Figure 16). The resulting cage complex 2,3,4,5,6,7,8-hepta(methoxycarbonyl)pentacyclo [7.5.0.0^2.4^.0^3.8^.0^5.14^]-tetradeca-7,10,12-triene (**48d**) was then treated with the electron-deficient dienophiles dimethyl acetylenedicarboxylate (DMAD) and maleimide, yielding [4 + 2] cycloaddition products in high yields. NMR and X-ray diffraction were used to conduct a thorough study [92].

The synthesis of crown compounds containing cycloheptatriene (**1a**) and the tropylium ion (**1b**) from 1,6-dithiocyanatocycloheptatriene was proposed by Okazaki [93]. He suggested the photochemical synthesis of 1,6-dithiocyanatocycloheptatriene from benzocyclopropene and thiocyanogen. The reaction of dithiolate (**49a**) with cis-dichloroethylene (**49b**) afforded products (**49c**), (**49d**), and (**49e**) in yields of 11%, 13%, and 11%, respectively. The cis orientation of the halide used in this reaction is responsible for the facile formation of the macrocycles (**49d**) and (**49e**). Moreover, di-iodide was utilized to synthesize sulfur-containing macrocycles [93].

Pursiainen demonstrated the synthesis of podands bearing aromatic end groups and complex formation with tropylium tetrafluoroborate **(1c)** in 1,2-dichloroethane [94]. π–π stacking and cation–π interactions are important in the host–guest complexation of benzene-substituted crown ethers with electron-deficient aromatic carbenium ions such as tropylium and pyridinium. The complexation behavior of the podands with tropylium tetrafluoroborate (**1c**) in 1,2-dichloroethane (DCE) was tracked by absorption changes in the UV–visible spectra. Podands were synthesized by Williamson ether synthesis, and their complexation with (**1c**) was observed by UV. The exceptional study of the author revealed that non-covalent intermolecular forces that lead to the complex development between podands and tropylium ions are cooperative interactions. Podands and their complexation ability have recently been used in supramolecular chemistry, most notably in the synthesis of rotaxanes and pseudorotaxanes. The study’s main goal was to find out how the macrocyclic effect, donor atoms, and aromatic substituents affected the complexation of aromatic cations [94].

Miquel Solà and Sílvia Escayola’s article explores double aromaticity in substituted tropylium cations [95]. Hexaiodobenzene and hexakis(phenylselenyl)benzene dications are known for their σ- and π-double aromaticity, making their characterization essential for organic and structural chemistry. Tropylium cations, with their 7 neutral halogen substituents, were expected to exhibit σ-aromaticity due to their 14 σ-electrons. However, the study found that the most stable geometries were highly puckered, leading to a loss of delocalization in both the π- and σ-systems. The only system that showed double aromaticity was triplet C_7_Br_7_^+3^, which had an internal Hückel aromatic tropylium ring and an external incipient Baird aromatic Br_7_ ring. However, this result was dependent on the functional used and revealed the need to balance various factors in designing double-aromatic molecules. The article discusses the requirements for double aromaticity, including electron counting corresponding to the Hückel or Baird rule and the presence of σ- and π-electron delocalization. The presence of strong electronic repulsion between external substituents or substituents with np orbitals that are not diffuse enough can quench the σ-aromaticity by puckering the benzene or tropylium cation rings. The most interesting species analyzed in the study were 1C_7_Br_7_^+3^, which had an internal Hückel aromatic tropylium ring and an external Hückel antiaromatic Br_7_ ring, and 3 C_7_Br_7_^+3^, which had an internal Hückel aromatic tropylium ring and an external weak Baird aromatic Br_7_ ring [95].

Andras Bodi conducted a study on the dissociation of energy-selected 1,3,5-cycloheptatriene (CHT) and toluene (Tol) cations using imaging photoelectron photoion coincidence spectroscopy [96]. The study found that in the measured energy ranges of 10.30–11.75 eV for CHT and 11.45–12.55 eV for Tol, only the hydrogen atom loss channels were observed, resulting in the formation of C_7_H_7_ ions from both molecular ions. These ions are metastable at the H-loss threshold. The decomposition of gaseous C_7_H_8_ ions is a widely studied ion reaction due to their prevalence in the mass spectra of aromatic compounds. Hydrogen abstraction from C_7_H_8_^+•^ ions produces more than 30 possible isomeric structures of C_7_H_7_ cations, with benzyl (Bz^+^), tropylium (Tr^+^), and tolyl (Tl^+^) being the most significant ones among them [96].

**Figure 17:**
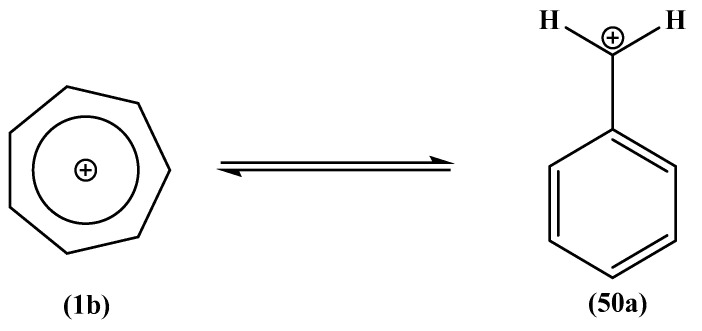
Benzylium and tropylium isomers of the C_7_H_7_^+^ cation.

In this article, Michael A. Duncan conducted a study on C_7_H_7_^+^ ions obtained from toluene and cycloheptatriene through electrical discharge in a supersonic expansion [97]. The infrared spectra of both isomers, benzylium (**50a**) and tropylium (**1b**), were studied using photodissociation and messenger tagging. The spectra were analyzed based on their argon tagging efficiency, relative abundance upon precursor change, vibrational frequency computations, and previous experimental studies. The benzylium isomer was more prominent with both precursors, while the tropylium isomer was more challenging to tag with argon, possibly due to its more exothermic formation. The nitrogen-tagged tropylium ion showed two bands at 3036 and 1477 cm^−1^ in the explored infrared region, and the previously known vibrational structure of the benzylium ion was extended to the C-H stretching region. The obtained spectra agreed with previous studies of the ions in HBr solutions and rare gas matrices. The study provided insights into the spectral properties of both isomers of C_7_H_7_^+^ and assigned them based on frequency computations and comparisons to previous experimental studies (Figure 17) [97].

**Figure 18:**
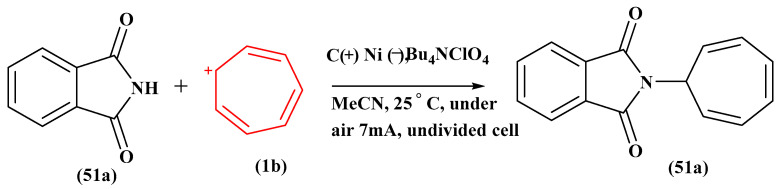
Cross-dehydrogenative couplings of C–H/N–H bonds by electrochemical oxidation.

Lifang Tian’s article details the anodic oxidation of 1,3,5-cycloheptatriene or xanthenes to produce the tropylium ion or xanthene cations [98], which are subsequently subjected to nucleophilic attack by azoles and imides to form C-N bonds. Electrochemical oxidation has been proposed as a sophisticated substitute for external chemical oxidants employed in C-N bond formation. The carbocations created at the anode are highly reactive species, referred to as “hot carbocations”, that can easily undergo reactions with mildly nucleophilic solvents and rearrangement or elimination to form alkenes. Therefore, to enable C-N bond formation, the carbocation intermediates must be sufficiently stabilized and long-lasting. This novel strategy is highly feasible due to the natural merits of the components: 1,3,5-cycloheptatriene can be easily oxidized into the aromatic, stable cycloheptatrienyl cation, tropylium, which can react with several nucleophiles to form a covalent bond. Xanthenes are vital motifs that are reported to be oxidized under electrochemical conditions to form stabilized diarylcarbenium ions. Azoles and imides are significant moieties in organic chemistry and typically serve as good nucleophiles. The desired product is formed (**51b**) with a yield of 92%, which is nearly identical to the yield under electrolysis (Figure 18). The tropylium ion (**1b**) and xanthene cations serve as key intermediates in forming the C-N bond and are highly electrophilic, directly attacked by azoles to deliver the C-N-coupled products [98].

The synthesis of a charge-transfer (CT) complex employing an aromatic carbonium tropylium (Tr^+^) and a macrocycle-based host–guest interaction is covered in Lihua Yuan’s study [99]. In order to control the properties of liquid crystals, the paper emphasizes the significance of host–guest interactions and underlines the special capacity of cyclo [6]aramides, a form of macrocyclic host with a shape-persistent backbone, to preferentially recognize various guests. The article also discusses how the extreme clustering of macrocycles in solvents makes it difficult to determine the binding affinity of the macrocycles toward guest molecules. The ability of cyclo [6]aramides containing linear alkyl chains to distinguish between loose and intimate dibutyl ammonium (DBuA) ion pairs with the naked eye is addressed in the article. It was discovered that cyclo [6]aramide, which was employed to solve the aggregation difficulty, forms a CT complex with tropylium tetrafluoroborate through a potent CT interaction and H-bonding. This is the first instance of host–guest chemistry involving Tr^+^ between macrocycles with hydrogen bonds and carbonium cations. Tr^+^ has been found to be interesting in host–guest chemistry with diverse kinds of macrocycles in earlier investigations [99].

Koichi Komatsu’s paper describes the synthesis and characteristics of tropylium ion derivatives with distinctive structures in a study that sought the highest carbocation stability feasible [100]. The work was carried out by the author’s group. Professor Tetsuo Nozoe’s discovery of hinokitiol and its unusual aromaticity, which is connected to tropolone, tropone, and the aromaticity of the tropylium ion, is one of the most important achievements in organic chemistry. The final thermodynamic stabilization of the hydrocarbon carbocation lacking any electronic alterations, such as heteroatom substitution, was of interest to the author. Professor Nozoe provided advice to the author on this matter. The study looked at brand-new techniques for cation stabilization, including through-space electron donation by an intramolecularly fixed-electron donor and electron donation to the electron-deficient system from bonds that are rigidly placed parallel to the cation 2p orbitals. The research demonstrates that, in two model cations with various carbon frameworks, the through-space electron transfer from a benzene ring arranged in a face-to-face configuration stabilizes the cation by nearly two pKR^+^ units. The study also shows that the tropylium ring can be annealed to a hard BCO unit, which is practically equivalent to substituting it with two cyclopropyl groups, to show the efficiency of conjugation and inductive electron donation. The hydrocarbon-derived tropylium ion with three BCO units and a pKR^+^ value of 13.0 is the most stable. The tropylium ion was also connected to acetylene or phenylene spacers in the study to prepare stable dications, which, following a one- or two-electron reduction, produced a stable radical cation or triplet diradical. A new technique for stabilizing the cationic system, annelation with BCO units, was discovered as a result of the investigation, which sought to develop a tropylium ion with high stability in the end. With little to no electronic disruption, as would happen in the event of structural alteration by heteroatom replacement, the features of the original system are preserved with this technique. This approach is anticipated to be expanded upon in order to find a number of fresh, stable electron-deficient complexes, ushering in a new era in fundamental research on carbocations [100].

Composite materials, which are made up of both organic and inorganic elements, have the potential to be useful materials with intriguing electrical and optical properties, according to a paper by James R. Neilson [101]. The production and structure of two hybrid substances with the optoelectronically reactive tropylium ion in inorganic tin- and lead-iodide systems are discussed in the study. Tropylium lead iodide is a vivid red-orange powder and features one-dimensional bands of face-sharing lead(II)-iodide octahedra, whereas tropylium tin iodide contains distinct tin(IV)-iodide octahedra. The two compounds’ different topologies, organic–inorganic interactions, and observable optoelectronic capabilities are all covered in the paper. While tropylium lead iodide was created by the action of tropylium tetrafluoroborate and PbI_2_ in aqueous hydroiodic acid, tropylium tin iodide was created by the reaction of SnI4 and tropylium iodide. The article’s recommendation for improving electronic coupling and carrier mobility is to look for tropylium-containing substances with higher inorganic dimensionality [101].

Ab initio density functional studies on the tropylium trication, (C_7_H_7_)^3+^, show an intact 3D hexagonal-pyramidal configuration with a carbon atom in the heptacoordinate state, based on a report by A.K. Fazlur Rahman [102]. It has been discovered that this substance is stable in the face of structural deformation, dissociation, and deprotonation. On the other hand, it is demonstrated that the pyramidal structures of ionic C_8_H_8_ compounds, which would include an octacoordinated carbon atom, are unstable. These discoveries provide important information for designing new hypercarbon-containing molecules that may be used for hydrogen storage and chemical synthesis. In comparison to the associated monocation and dication, the tropylium trication, (C_7_H_7_)^3+^, exhibits more distinguishing characteristics. Its heptacoordinate carbon atom is part of a stable three-dimensional pyramidal structure, and even after the configuration is optimized, the pyramidal form remains unchanged. The pyramidal shape of (C_7_H_7_)^3+^ is more energetically stable because its total energy is less than that of the analogous 2D ring configuration. The ionic hydrocarbon tropylium trication (C_7_H_7_)^3+^ can contain heptacoordinate carbon, as shown by the ab initio DFT calculations. The 3D hexagonal-pyramidal (C_7_H_7_)^3+^, which has a carbon atom in the heptacoordinate state, is projected to have a metastable configuration that is relatively stable against structural alterations and very stable against the dissociation of CH^+^. The 4n + 2 interstitial electron criteria for 3D aromaticity provides an explanation for these findings. Conversely, the previously postulated hexagonal-pyramidal heptamethylbenzene, (C_7_H_7_)^3+^, and the tropylium monocation and dication, (C_7_H_7_)^+^ and (C_7_H_7_)^2+^, do not contain heptacoordinate carbon, and neither of the ionic C_8_H_8_ substances involves octacoordinated carbon. It is demonstrated that each of the suggested heptagonal-pyramidal configurations is unstable. These findings give important information for creating new hypercarbon-containing molecules that could be used for hydrogen storage and chemical synthesis [102].

The two competing isomers, cationic benzylium and tropylium, which can come from the dehydrogenation of methylated polycyclic aromatic hydrocarbon (PAH) cations and are anticipated to be the two lowest-energy isomers, are addressed in Christine Joblin’s paper [103]. The dominant isomer was identified via IR predissociation spectroscopy, with the greatest depletion band around 1620 cm^−1^ suggesting the prevalence of the benzylium-like isomers. To show the spectral characteristics of the two lowest-energy isomers, XCH^+2^ and XC^+7^, as well as the species produced in the dissociative ionization of methylated PAHs, synthetic spectra were created. Depletion measurements revealed that only one isomer was found in the more compact PAH, C_17_H_11_, whereas at least two isomers appeared in considerable fractions among species produced from acene. Due to a reduced isomerization energy barrier, it was discovered that tropylium-like isomers were more common in species produced from acenes. However, tropylium-like isomers are not anticipated to be present in astrophysical environments where methylated PAHs are exposed to UV photons, with the exception of small cat-condensed PAHs at a maximum relative abundance of about 40%. The study suggests that XCH^+2^ species are appealing candidates to account for the 6.2 m AIB. Even for more compact PAHs, additional isomerization caused by exposure to ultraviolet (UV) rays may result in certain tropylium-like isomers [103].

**Figure 19:**
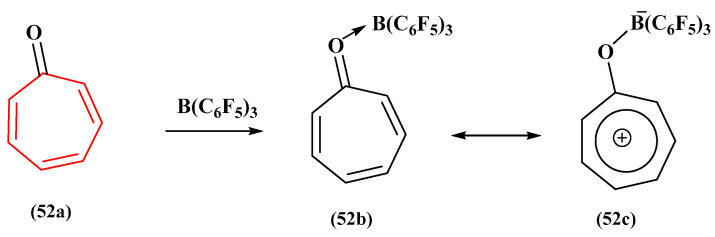
Synthesis of a new family of tropylium ions: tropone–triarylborane complexes.

By treating tropones (**52a**) in a 1:1 ratio with B(C_6_F_5_)^3^, Takeshi Fujita and Junji Ichikawa proposed an approach to yield stable tropone–triarylborane complexes (**52c**) in high yields (Figure 19) [104]. The quantity of fused benzene rings determines how aromatic the resultant complexes are, and they have betaine structures made up of tropylium and borate moieties. The investigators sought a viable method for the use of stable tropyliums as optical materials and discovered that B(C_6_F_5_)_3_ was the best Lewis acid for the job. The authors created air- and thermally stable tropone-B(C_6_F_5_)_3_ complexes with two or fewer fused benzene rings by combining tropones and B(C_6_F_5_)_3_ in an equimolar ratio. The complexes’ betaine structures were validated by spectroscopic studies and DFT calculations, which also revealed that complexes with fewer fused benzene rings had more aromaticity. This study offers a possible path toward creating practical materials using stable tropylium ions [104].

**Figure 20:**
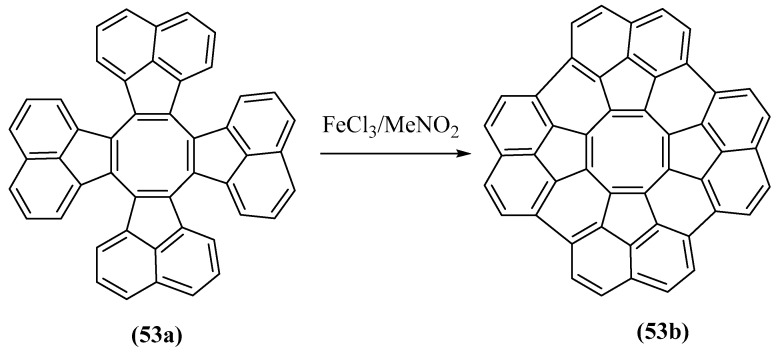
Synthetic attempt to make nanographene (**53b**) from compound (**53a**).

In their article, Naoki Aratani and Hiroko Yamada presented the conversion of non-aromatic COT (**53a**) into a cycloheptatriene spirolactone upon oxidation, which could be reversibly interconverted to the aromatic tropylium cation by acid/base treatment [105]. This conversion of COT was achieved through both chemical and electrochemical methods, and further oxidation of cycloheptatriene produced benzene. This process resulted in a reduction in the π-conjugated ring size, which enhanced the aromatization. This transformation has implications for the structural stability from the perspective of the aromatic characteristics. The oxidation of non-aromatic COT, a non-planar compound, resulted in the formation of the aromatic tropylium cation (**53b**), which is formally stable, accompanied by a carbon-skeleton rearrangement. The cation radical was first generated by oxidizing the compound with FeCl_3_, which then reacted with molecular oxygen. Rearrangement occurred, as illustrated, followed by dioxirane formation–decomposition that generated cycloheptatriene with spirolactone (Figure 20) [105].

In this paper, Raakhi Gupta examines the structural and aromatic properties of conventional carbocyclic and heterocyclic systems after switching CH moieties with phosphorus atoms (CH/P) [106]. Additionally, there have been attempts to analytically and physically investigate the idea of the carbon copy for the phosphorus atom in its low coordination state. The tropylium ion is a widely recognized non-benzenoid aromatic system whose aromaticity has been well investigated. Gupta used a number of variables, including bond equalization, aromatic stabilization energies (ASE), nucleus-independent chemical shift (NICS) values, proton nuclear magnetic resonance (^1^H-NMR) chemical shifts, magnetic susceptibility exaltation, and magnetic anisotropy values, to investigate the impact of mono- and poly-CH/P exchange on the aromaticity of the tropylium ion. The ASE values for the mono- and diphospha analogs range from −46.3 kcal/mol to −6.2 kcal/mol, showing their aromatic nature. Ions with three and four phosphorus atoms, however, start to lose their planarity, and their ASE values start to resemble those of non-aromatic structures. The NICS(1)zz values for all of the systems under study are negative, which is compatible with their aromatic nature. The aromatic area also corresponds to the ^1^H-NMR chemical changes. Their aromaticity is further supported by the energy difference between their highest occupied molecular orbital (HOMO) and lowest unoccupied molecular orbital (LUMO), which varies from 3 × 10^−19^ to 9 × 10^−19^ J. Systems with more than four phosphorus atoms cannot maintain their monocyclic structures. The aromaticity of the tropylium ion is maintained for a maximum of four carbon atoms upon CH/P exchange. However, the system transforms into bicyclic and polycyclic structures with additional exchange(s). The magnetic field effect’s markers, such as ^1^H-NMR chemical shifts, NICS(1)zz, magnetic susceptibility exaltation, and magnetic anisotropies, repeatedly demonstrate that these species are aromatic [106].

**Figure 21:**
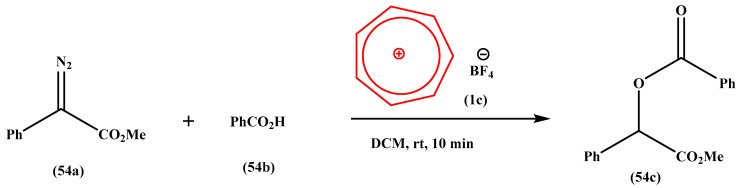
The role of homoaromaticity in the tropylium-catalyzed carboxylic acid OH insertion with diazoesters.

A new process for the tropylium-catalyzed carboxylic acid O-H insertion with diazoesters (**54a**) to create hydroxy esters (**54c**) is described by Anakuthil Anoop and Venkataraman Ganesh in their paper (Figure 21) [107]. Instead of the activated carbene that served as the essential intermediate in the prior method, their computer version suggests a distinct homoaromatic intermediate that includes the tropylium ion and the diazoester. To investigate the susceptibility of their model, the energy profiles were assessed using several computational techniques. They took into account five alternative pathways, including the suggested activated carbene pathway, the homoaromatic intermediate pathway, concerted O-H insertion by the carbene, and protonation/nucleophilic attack on the diazoester, for the tropylium-ion-catalyzed O-H insertion of diazo compounds. They were able to further investigate the breakdown of diazoesters with the tropylium ion, followed by an SN_2_ attack involving the homoaromatic intermediate, thanks to their computational model’s discovery of the homoaromatic character of the C-bound tropylium carbene intermediate. The model validates the experimental findings in terms of the substrate scope and energy barrier at room temperature because the modeled reaction profile was extremely energetically beneficial, in contrast to the other ways taken into account [107].

In this article, the progress of a formal phenylation reaction was disclosed by Thanh Vinh Nguyen [108]. This reaction proceeds through an electrophilic cycloheptatrienylation with the tropylium ion, followed by an oxidative ring contraction. Recent endeavors have been directed toward the creation of transition-metal-free aryl–aryl coupling methods, as highly toxic reagents and harsh reaction conditions have been observed. The synthetic precursors for such reactive intermediates typically require lengthy synthetic sequences, and their high reactivity often results in undesired side reactions. Therefore, there is significant interest in developing a fresh synthetic approach for the phenylation reaction. One such approach involves performing an oxidative ring-contraction reaction on the electron-deficient seven-membered ring of the tropylium (-C_7_H_6_^+^) ion, which can transform it into a phenyl ring (-C_7_H_6_).

**Figure 22:**
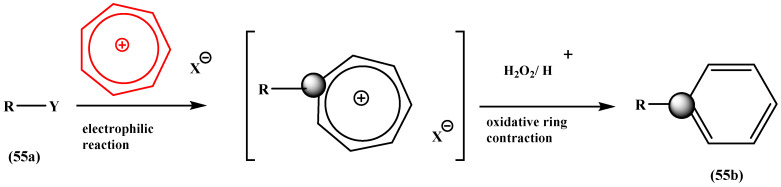
Formal electrophilic phenylation reaction with tropylium ion.

Our proposed technique offers a fresh approach for the electrophilic installation of a phenyl group without the use of transition-metal catalysis, which could be of immense significance in synthetic chemistry [108]. Therefore, the transformation from a tropylium ion to a phenyl group via ring contraction should be energetically feasible. Through extensive optimization studies, we determined that the ideal reaction conditions involve utilizing an aqueous/acetonitrile environment with H_2_O_2_ (3 equiv.) as the oxidant, along with the presence of HBF_4_ (2 equiv.), which results in an exceptional yield of 93%. In a transition-metal-free fashion, we have created a fresh approach to conducting formal phenylation reactions of aryl halides (**55a**) and electron-rich arenes. This method utilizes the versatile electrophilicity and oxidizing capability of the tropylium ion (**1c**) to create the seven-membered ring framework and then contract one carbon atom from it to generate the phenyl ring (Figure 22) (**55b**). At a fundamental level, this transformation is intriguing since the non-benzenoid aromatic tropylium ring is altered into the aromatic benzene ring [108].

**Figure 23:**
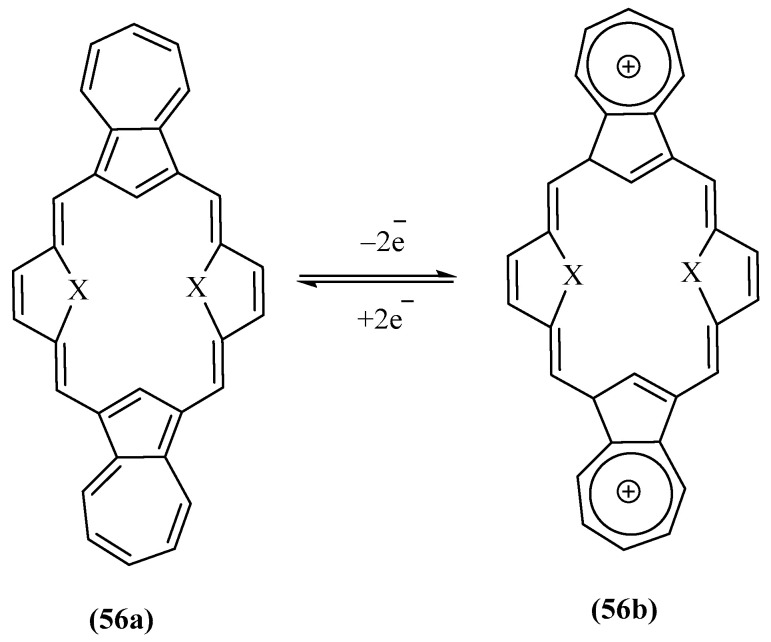
The delocalization in diheterodiazuliporphyrins frames (X = NH, O or S).

Using the topological resonance energy (TRE) method, Tuerhong, M., and Kerim, A., investigated the aromaticity of a series of diheterodiazuliporphyrins in both neutral (**56a**) and dicationic states (**56b**) [109]. The TRE results indicated that all isomers of diheterodiazuliporphyrins exhibited greater aromaticity in the dicationic states compared to their corresponding neutral states. They also evaluated the local aromaticity and the primary aromatic conjugation pathways through the bond resonance energy (BRE) and circuit resonance energy (CRE) indices for both states. The results indicated that the local aromaticity resulting from the small rings played a greater role in contributing to global aromaticity than the main aromatic conjugation pathway. In the neutral states, the azulene units were the primary contributors to global aromaticity, and the compounds were primarily stabilized by their 10π-electron peripheral structures. However, in their dicationic states, the main contributors to global aromaticity were the 6π-electron tropylium unit structures [109] (Figure 23).

The tropylium ion and its derivatives extend their applications in host–guest chemistry and polymerization chemistry. In host–guest chemistry, the tropylium ion serves as a guest molecule in supramolecular chemistry and can form inclusion complexes with host molecules. These complexes may serve as catalysts or as building blocks for the synthesis of novel materials. The formation of charge-transfer (CT) complexes from 2,6-helic [6]arene hosts and a tropylium cation is one example of the host–guest chemistry of the tropylium ion. It was discovered that a redox stimulus could efficiently control the binding and release processes of the guest in the complexes, and the response process could be visually monitored by the color change of the solution. This was the first observable redox-stimulus-responsive host–guest system composed of tropylium cations [110].

The tropylium ion can be utilized as a monomer in polymerization chemistry to synthesize polymers. Polytropylium can be formed by the polymerizing tropylium ion using anion initiators such as sodium naphthalenide. Poly(tropylium) is an electrically conductive polymer that could potentially be used in electronic devices. Tropylium derivatives can also be employed in the chemistry of polymerization. Tropylium derivatives with functional groups such as esters or amides, for example, can be polymerized to generate polymers with specified characteristics.

The tropylium ion and its derivatives have several potential uses in host–guest and polymerization chemistry. Due to their distinct chemical characteristics, they serve as interesting building blocks for the synthesis of novel materials with tailored properties.

## 9. Conclusions

The current review focuses on various organic synthesis reactions assisted by tropylium ions. The tropylium ion will continue to thrive as a versatile reagent capable of new synthetic transformations. It is a valuable building component in a variety of synthesis reactions, including one-pot chemical synthesis and medicinal chemistry. It is a promising candidate for avoiding noxious byproducts, making it useful in metal-free synthesis and therefore playing a role in green chemistry. Despite the numerous advantages of the chemistry promoted by the tropylium ion, there are some challenges to its widespread application in organic synthesis. The primary drawback is the expensive precursors to tropylium salts, making them less appealing as a new synthetic option. Alternative routes that use less expensive starting materials can be explored to overcome this challenge. Tropylium salts, for example, can be synthesized by reacting cycloheptatriene with sulfur trioxide, which is a less costly alternative to employing tropylium halides as precursors. Another low-cost alternative approach utilizes the reaction of phenylacetylene or acenaphthylene with Lewis acids. Furthermore, tropylium halides are relatively unstable in the presence of air and moisture. Consequently, they are often prepared in situ using a careful selection of solvents and co-reagents to avoid side reactions. Tropylium halides’ instability can be minimized by performing reactions under inert conditions, such as nitrogen or argon. This can assist in preventing the production of undesirable byproducts as a consequence of exposure to air or moisture. To avoid the instability induced by halide ions, preformed tropylium salts can be utilized instead of tropylium halides. Tropylium tetrafluoroborate (C_7_H_7_BF_4_), for example, is more stable than tropylium chloride (C_7_H_7_Cl). Nevertheless, the tropylium ion and its derivatives can certainly be utilized further in host–guest and polymerization chemistry. One of the key advantages of tropylium ions in flow chemistry is their ability to act as highly reactive electrophiles, enabling rapid and efficient reactions with a wide range of nucleophiles. Additionally, tropylium ions are highly stable and can be generated in situ from readily available precursors, making them an attractive option for use in continuous-flow reactions. Overall, tropylium ions continue to play a crucial role in the advancement of modern chemical technologies, and their unique properties make them a valuable tool for synthetic chemists working toward the development of new and innovative chemical processes and materials.

## Data Availability

Not applicable.
